# Supplementary figures and images for: Germline and somatic albinism variants in amelanotic/hypomelanotic melanoma: Increased carriage of TYR and OCA2 variants
Source: PLoS One. 2020 Sep 23;15(9):e0238529. doi: 10.1371/journal.pone.0238529 (PMC7510969; doi:10.1371/journal.pone.0238529)

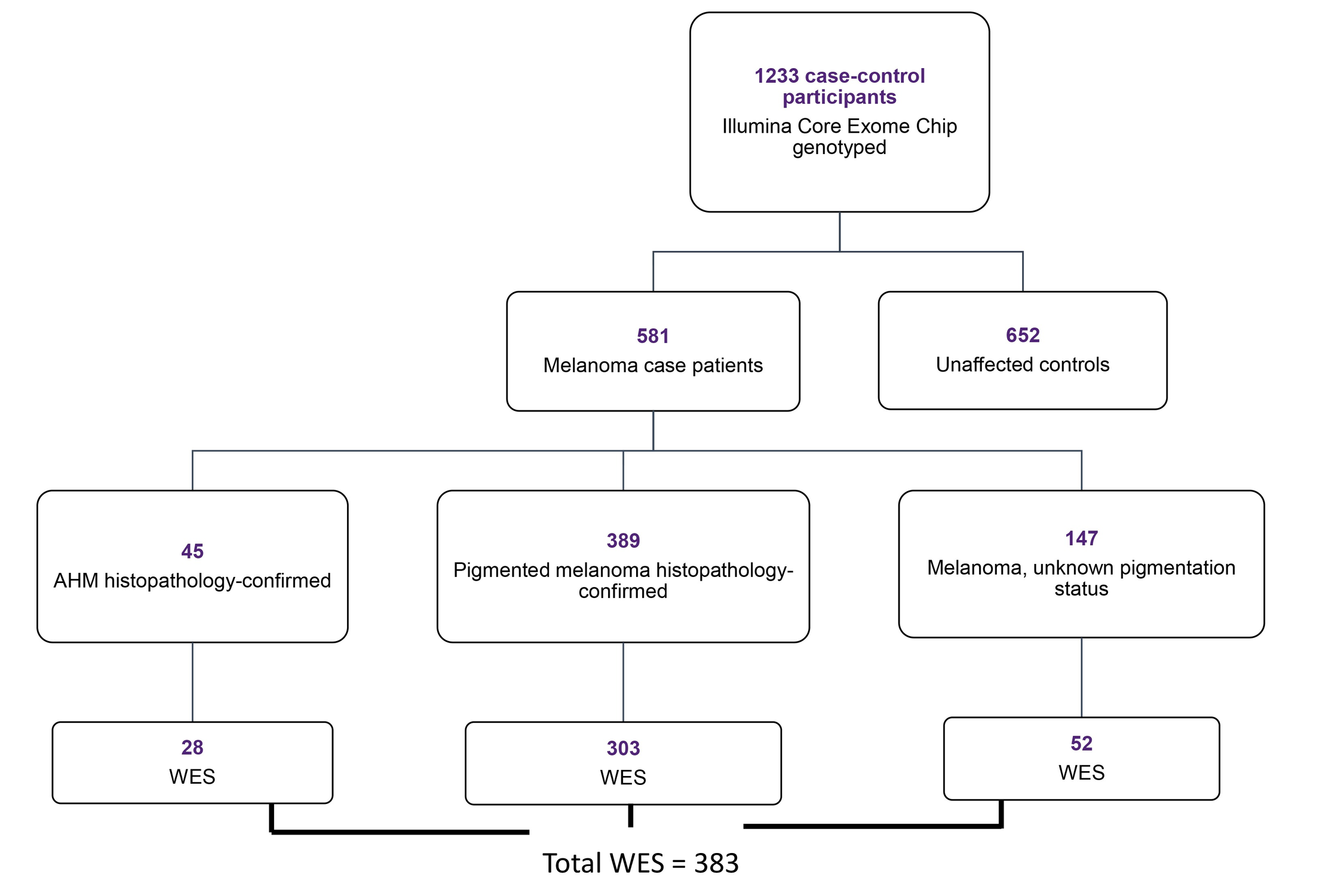

Supplement: S1 Fig — AHM amelanotic/hypomelanotic melanoma; WES whole exome sequencing. (TIF) [file pone.0238529.s001.tif]
