# Supplementary material for: Germline and somatic albinism variants in amelanotic/hypomelanotic melanoma: Increased carriage of TYR and OCA2 variants
Source: PLoS One. 2020 Sep 23;15(9):e0238529. doi: 10.1371/journal.pone.0238529 (PMC7510969; doi:10.1371/journal.pone.0238529)
Supplement: S1 Table — (DOCX) [file pone.0238529.s002.docx]

**Table S1: *TYR* and *OCA2* gene alleles in pigmented melanoma and amelanotic/hypomelanotic melanoma patients**

|  |  |  |  | **Controls** | | **Melanoma Cases** | | | ***X^2^* Statistical Tests (*P*-value)^u^** | | |
| --- | --- | --- | --- | --- | --- | --- | --- | --- | --- | --- | --- |
| **Gene/OCA** | **Chr:Position (**GRCh37) | **HGVS Transcript Variant** | **gnomAD ^a^**  **(MAF%)** | **MGRB** ^b^  **Control**  **N (MAF%)**  **Total = 1144**  **WES** | **BNMS** ^c^  **Control**  **N (MAF %)**  **Total = 652** ^d^ | **PM**  **N (MAF%)**  **Total = 389** ^d^  **(WES = 303)** | **AHM**  **N (MAF%)**  **Total = 45** ^d^  **(WES = 28)** | **Total Melanoma**  **Cases**  **N (MAF %)**  **Total = 581** ^b^  **(WES = 383)** | **Total Melanoma Cases vs MGRB + BNMS Controls** | **AHM vs PM Cases** | **AHM Case vs MGRB + BNMS Controls** |
| ***TYR/*OCA1** |  |  |  |  |  |  |  |  |  |  |  |
| rs28940881*A/G p.M1V ^g,h^ | 11:88911122 | NM_000372.4:c.1A>G | 0.012 | 1 (0.04) | 0 (0.0) ^d^ | 0 (0.0) ^d^ | 0 (0.0) ^d^ | 0 (0.0) ^d^ | 0.400 | 0.540 | 0.308 |
| rs61753178*C/T p.P21S ^f,g,h^ | 11:88911182 | NM_000372.4:c.61C>T | 0.0078 | 1 (0.04) | - | 0 (0.0) | 0 (0.0) | 0 (0.0) | 0.513 | 0.377 | 0.400 |
| rs1373014646*G/A p.A23T ^e^ | 11:88911188 | NM_000372.4:c.67G>A | 0.0018 | 0.0 | - | 0 (0.0) | 1 (1.8) | 1 (0.09) | **0.047** | **0.008** | **0.006** |
| rs28940876*C/T p.P81L ^f,g,h^ | 11:88911363 | NM_000372.4:c.242C>T | 0.017 | 1 (0.04) | - | 0 (0.0) | 0 (0.0) | 0 (0.0) | 0.408 | 0.500 | 0.385 |
| [rs759163678*G/C](http://www.ncbi.nlm.nih.gov/projects/SNP/snp_ref.cgi?rs=rs759163678) p.G106R ^g^ | 11:88911437 | NM_000372.4:c.316G>C | 0.0 | 1 (0.04) | - | 0 (0.0) | 0 (0.0) | 0 (0.0) | 0.526 | 0.488 | 0.323 |
| rs200471520*G/A p.G154E ^e,g^ | 11:88911582 | NM_000372.4:c.461G>A | 0.0017 | 1 (0.04) | - | 0 (0.0) | 0 (0.0) | 0 (0.0) | 0.526 | 0.540 | 0.392 |
| [rs1160771486*A/G](http://www.ncbi.nlm.nih.gov/projects/SNP/snp_ref.cgi?rs=rs1160771486) p.M179V ^e^ | 11:88911656 | NM_000372.4:c.535A>G | 0.0031 | 1 (0.04) | - | 0 (0.0) | 0 (0.0) | 0 (0.0) | 0.385 | 0.606 | 0.308 |
| rs1042602*C/A p.S192Y | 11:88911696 | NM_000372.4:c.575C>A | *36.4* | 855 (*37.4*) | 469 (*35.9*) ^d^ | 278 (*36.4*) ^d^ | 27 (*30.0*) ^d^ | 421 (*36.2*) ^d^ | 0.571 | 0.238 | 0.194 |
| rs61754362*C/A p.P205T ^f,g,h^ | 11:88911734 | NM_000372.4:c.613C>A | 0.0054 | 1 (0.04) | - | 0 (0.0) | 0 (0.0) | 0 (0.0) | 0.513 | 0.714 | 0.298 |
| rs61754365*G/A p.R217Q ^f,h^ | 11:88911771 | NM_000372.4:c.650G>A | 0.061 | 2 (0.09) | 0 (0.0) ^d^ | 1 (0.13) ^d^ | 0 (0.0) ^d^ | 1 (0.09) ^d^ | 0.769 | 0.425 | 0.714 |
| [rs758115945*G/A](http://www.ncbi.nlm.nih.gov/projects/SNP/snp_ref.cgi?rs=rs758115945) p.E221K ^g,h^ | 11:88911782 | NM_000372.4:c.661G>A | 0.0009 | 1 (0.04) | - | 0 (0.0) | 0 (0.0) | 0 (0.0) | 0.364 | 0.556 | 0.351 |
| rs61754368*GT p.K243fs ^e^ | 11:88911850 | NM_000372.4:c.729G>T | N/A | 1 (0.04) | - | 0 (0.0) | 0 (0.0) | 0 (0.0) | 0.444 | 0.571 | 0.328 |
| rs104894314*G/T p.V275F ^f,g,h^ | 11:88924373 | NM_000372.4:c.823G>T | 0.022 | 1 (0.04) | 1 (0.08) ^d^ | 3 (0.39) ^d^ | 0 (0.0) ^d^ | 4 (0.34) ^d^ | **0.022** | 0.073 | 0.690 |
| rs200854796*C/T p.R298W ^e^ | 11:88924442 | NM_000372.4:c.892C>T | 0.006 | 0.0 | - | (0.0) | (0.0) | 1 (0.09) | **0.049** | 0.114 | 0.741 |
| rs61754375*G/A p.R299H ^f,g,h^ | 11:88924446 | NM_000372.4:c.896G>A | 0.0054 | 0.0 | 1 (0.08) ^d^ | 1 (0.13) ^d^ | 0 (0.0) ^d^ | 1 (0.09) ^d^ | 0.103 | 0.134 | 0.800 |
| rs61754387*A/C p.N371T ^g,h^ | 11:88961066 | NM_000372.4:c.1112A>C | 0.0018 | 0.0 | - | 1 (0.17) | 0 (0.0) | 0 (0.0) | 0.058 | 0.094 | 0.909 |
| rs61754388*C/A p.T373K ^f,g,h^ | 11:88961072 | NM_000372.4:c.1118C>A | 0.068 | 0.0 | 2 (0.15) ^d^ | 2 (0.26) ^d^ | 1 (1.1) ^d^ | 3 (0.26) ^d^ | 0.055 | 0.091 | **0.020** |
| rs1126809*G/A p.R402Q | 11:89017961 | NM_000372.4:c.1205G>A | *27.28* | 667 (*29.15*) | 411 (*31.5*) ^d^ | 248 (*32.6*) ^d^ | 38 (*42.2*) ^d^ | 373 (*32.1*) ^d^ | 0.066 | 0.070 | **0.015** |
| rs104894313*C/T p.P406L ^f,g,h^ | 11:89017973 | NM_000372.4:c.1217C>T | 0.43 | 14 (0.598) ^i^ | - | 4 (0.66) | 1 (1.8) | 5 (0.67) | 0.796 | 0.069 | 0.310 |
| rs543973275*TT p.T489fs ^e^ | 11:89028407 | NM_000372.4:c.1463T= | N/A | 0.0 | - | (0.0) | (0.0) | 1 (0.09) | 0.063 | 0.571 | 0.769 |
| rs1042602*A p.192Y- rs1126809*A p.402Q |  |  | *1.9* ^j^ |  | (*1.8*) | (*2.1*) | (*2.8*) | (*2.2* SPM ^m^) (*2.9* MPM) |  | 0.28 |  |
| **Allelic spectrum for *TYR* in each subgroup** |  |  | 17 of 20 | 14 of 20 | 5 of 7 | 8 of 20 | 5 of 20 | 10 of 20 |  |  |  |
| **Allelic spectrum for *TYR* (MAF<1%) in each subgroup** |  |  | 15 of 18 | 12 of 18 | 3 of 5 | 6 of 18 | 3 of 18 | 8 of 18 |  |  |  |
|  |  |  |  | **Controls** | | **Melanoma Cases** | | | **SMMAT Tests (*P*-value)** | | |
| **Total number of *TYR* alleles (combined MAF<1%** ^s^**) observed in each subgroup** |  |  |  | 26 (1.14) | 4 (0.31) | 12 (1.76) | 3 (4.67) | 17 (2.22) | 0.065 ^q^ | **0.0088 ^q^** | **1.7x10^-14^ ^q^** |
|  |  |  |  | **Controls** | | **Melanoma Cases** | | | ***X^2^* Statistical Tests (*P*-value)^u^** | | |
| ***OCA2/*OCA2** |  |  |  |  |  |  |  |  |  |  |  |
| rs1254334474*-/C p.A55fs ^f^ | 15:28326857 | NM_001300984.1:c.163dup | 0.0015 | 0.0 | - | 1 (0.17) | 0 (0.0) | 1 (0.09) | 0.071 | 0.137 | 0.625 |
| rs147785669*T/A p.R76W ^e^ | 15: 28326795 | NM_000275.2:c.226A>T | 0.015 | 1 (0.04) | 0 (0.0) ^d^ | 0 (0.0) ^d^ | 0 (0.0) ^d^ | 0 (0.0) ^d^ | 0.588 | 0.488 | 0.476 |
| rs190612616*G/A p.P211L ^f,g,I,k^ | 15:28267661 | NM_001300984.1:c.632C>T | 0.005 | 0.0 | 0 (0.0) ^d^ | 1 (0.13) ^d^ | 0 (0.0) ^d^ | 1 (0.09) ^d^ | 0.055 | 0.063 | 0.690 |
| rs33929465*G/A p.R266W ^f,l,n^ | 15: 28263554 | NM_001300984.1:c.796C>T | 0.17 | 7 (0.31) | 3 (0.23) ^d^ | 0 (0.0) ^d^ | 0 (0.0) ^d^ | 1 (0.09) ^d^ | - | - | - |
| rs1800401*G/A p.R305W | 15:28260053 | NM_001300984.1:c.913C>T | *5.05* | 105 (*4.59*) | 75 (*5.75*) ^d^ | 38 (*4.97*) ^d^ | 3 (*3.33*) ^d^ | 54 (*4.65*) ^d^ | 0.540 | 0.408 | 0.317 |
| rs142931246*T/C p.Y342C ^f,h^ | 15:28259941 | NM_001300984.1:c.1025A>G | 0.047 | 4 (0.17) | 0 (0.0) ^d^ | 0 (0.0)^d^ | 0 (0.0) ^d^ | 0 (0.0) ^d^ | 0.247 | 0.870 | 0.278 |
| [rs34731820*A/G](http://www.ncbi.nlm.nih.gov/projects/SNP/snp_ref.cgi?rs=rs34731820) p.I370T ^e,g,k,p^ | 15:28235729 | NM_000275.2:c.1109T>C | 0.00077 | 0.0 | 0 (0.0) ^d^ | 1 (0.13) ^d^ | 0 (0.0) ^d^ | 1 (0.09) ^d^ | 0.053 | 0.059 | 0.741 |
| rs150335311*G/A p.T387M ^f^ | 15:28234769 | NM_000275.2:c.1160C>T | 0.0031 | 1 (0.04) | 0 (0.0) ^d^ | 0 (0.0) ^d^ | 0 (0.0) ^d^ | 0 (0.0) ^d^ | 0.606 | 0.667 | 0.667 |
| rs1800407*C/T p.R419Q | 15:28230318 | NM_000275.2:c.1256G>A | *6.5* | 198 (*8.65*) | 116 (*8.9*) ^d^ | 79 (*10.34*) ^d^ | 11 (*12.22*) ^d^ | 125 (*10.76*)^d^ | 0.080 | 0.556 | 0.488 |
| rs121918166*C/T p.V443I ^f,g,h,o^ | 15:28230247 | NM_000275.2:c.1327G>A | 0.51 | 25 **(***1.09***)** | 11 (0.84) ^d^ | 12 (*1.57*) ^d^ | 4 (*4.44*) ^d^ | 21 (*1.81*) ^d^ | **0.041** | 0.093 | **0.010** |
| rs121918170*T/C p.N489D ^f,g,h^ | 15:28228529 | NM_000275.2:c.1465A>G | 0.067 | 2 (0.09) | 3 (0.23) ^d^ | 1 (0.13) ^d^ | 0 (0.0) ^d^ | 1 (0.09) ^d^ | 0.741 | 0.364 | 0.278 |
| rs202126510*T/C p.N495D ^e^ | 15:28228511 | NM_000275.2:c.1483A>G | 0.004 | 1 (0.04) | - | 0 (0.0) | 0 (0.0) | 0 (0.0) | 0.465 | 0.556 | 0.333 |
| [rs755768280](http://www.ncbi.nlm.nih.gov/projects/SNP/snp_ref.cgi?rs=rs755768280)*G/A p.H615Y ^e^ | 15:28197038 | NM_000275.2:c.1843C>T | 0.0035 | 2 (0.09) | - | 0 (0.0) | 0 (0.0) | 0 (0.0) | 0.323 | 0.571 | 0.476 |
| [rs775872950*CA/C](http://www.ncbi.nlm.nih.gov/projects/SNP/snp_ref.cgi?rs=rs775872950) p.F685fs ^b,c,h^ | 15:28171296 | NM_000275.2:c.2055del | 0.0044 | 0.0 | - | 1 (0.13) | 0 (0.0) | 1 (0.09) | 0.061 | 0.146 | 0.741 |
| [rs753088699](http://www.ncbi.nlm.nih.gov/projects/SNP/snp_ref.cgi?rs=rs753088699)*G/A p.A709V ^e^ | 15:28117022 | NM_000275.2:c.2126C>T | 0.0026 | 1 (0.04) | - | 0 (0.0) | 0 (0.0) | 0 (0.0) | 0.556 | 0.588 | 0.454 |
| [rs768934658*A/C](http://www.ncbi.nlm.nih.gov/projects/SNP/snp_ref.cgi?rs=rs768934658) p.L734R ^e,k,o^ | 15:28116343 | NM_000275.2:c.2201T>G | 0.00088 | 0.0 | - | 1 (0.13) | 0 (0.0) | 1 (0.09) | 0.071 | 0.150 | 0.769 |
| rs374578342*A/G p.M769T ^b,c^ | 15:28096560 | NM_000275.2:c.2306T>C | 0.0039 | 1 (0.04) | - | 0 (0.0) | 0 (0.0) | 0 (0.0) | 0.425 | 0.465 | 0.377 |
| [rs776814755](http://www.ncbi.nlm.nih.gov/projects/SNP/snp_ref.cgi?rs=rs776814755)*C/T p.C777Y ^e,f,h^ | 15:28096536 | NM_000275.2:c.2330G>A | 0.0044 | 1 (0.04) | - | 0 (0.0) | 0 (0.0) | 0 (0.0) | 0.588 | 0.571 | 0.408 |
| **Allelic spectrum for *OCA2* in each subgroup** |  |  | 18 of 18 | 13 of 18 | 5 of 10 | 9 of 18 | 3 of 18 | 10 of 18 |  |  |  |
| **Allelic spectrum for *OCA2* (MAF<1%) in each subgroup** ^t^ |  |  | 15 of 15 | 10 of 15 | 3 of 7 | 6 of 15 | 0 of 15 | 7 of 15 |  |  |  |
|  |  |  |  | **Controls** | | **Melanoma Cases** | | | **SMMAT Tests (*P*-value)** | | |
| **Total number of *OCA2* alleles (combined MAF<1%** ^s^**) observed in each subgroup** ^t^ |  |  |  | 21 (0.90) | 6 (0.45) | 6 (0.95) | 0 (0.00) | 7 (0.91) | 0.61 ^r^ | 0.81 ^r^ | 0.84 ^r^ |
| ***TYR/*OCA1 *+ OCA2/*OCA2** |  |  |  |  |  |  |  |  |  |  |  |
| **Allelic spectrum for *TYR* and *OCA2* in each subgroup** |  |  |  | 27 of 38 | 10 of 17 | 17 of 38 | 8 of 38 | 20 of 38 |  |  |  |
| **Allelic spectrum for *TYR* and *OCA2* (MAF<1%) in each subgroup ^t^** |  |  |  | 22 of 33 | 6 of 12 | 12 of 33 | 3 of 33 | 15 of 33 |  |  |  |
| **Total number of *TYR* and *OCA2* alleles (combined MAF<1%** ^s^**) observed in each subgroup** ^t^ |  |  |  | 47 (2.04) | 10 (0.76) | 18 (2.71) | 3 (4.67) | 24 (3.13) | **0.008** | 0.90 | 0.095 |

^a^ Lek et al., 2016 [39] <http://exac.broadinstitute.org>. European Non-Finnish, listing the minor allele frequency.

^b^ Lacaze et al., 2019 [33], The Medical Genome Reference Bank

^c^ Duffy et al., 2020 [22]

^d^ Illumina Core Exome genotyping

^e^ Deleterious by *in silico* analysis using Polyphen2 [30] or MutationTaster [32], with all prediction tools shown in Supplementary File 5

^f^ Pathogenic albinism variant reported by Lasseaux et al., 2018 [3]

^g^ Albinism database <http://www.ifpcs.org/albinism/oca1mut.html>, albinism allele

^h^ Clinical Significance assigned as pathogenic or likely pathogenic in NCBI ClinVar database

^i^ Calculated from the frequency given for rs104894313 in 2288 MGRB genomes (sgc.garvan.org.au/initiatives/mgrb)

^j^ Jagirdar et al., 2014 [40]

^k^ Clinical Significance assigned as uncertain in NCBI ClinVar database

^l^ Clinical Significance assigned as benign in NCBI ClinVar database

^m^ SPM, single primary melanoma; MPM, multiple primary melanoma

^n^ gnomAD African population frequency 2.6%

^o^ OCA2 albinism allele Hawkes et al., 2013 [43]

^p^ Albinism database <http://www.ifpcs.org/albinism/oca1mut.html>, polymorphism

^q^ SMMAT *P*-value for comparison between BNMS WES cases and MGRB WES controls (excluding BNMS array controls), TYR: **0.0002**, 0.10, **1.1x10^-5^**

^r^ SMMAT *P*-value for comparison between BNMS WES cases and MGRB WES controls (excluding BNMS array controls), OCA2: 0.46, 0.67, 0.94.

^s^ Genotypes calculated as “missing completely at random” with respect to disease status and % then appropriately weighted for each denominator of the variants being summed.

^t^ Does not include rs121918166*C/T p.V443I

^u^ Bonferroni corrected (three phenotypes x 30 variants) critical *P=*0.0005 equivalent to a table wide α=0.05

AHM, amelanotic/hypomelanotic melanoma

BNMS, Brisbane Naevus Morphology Study

GRCh37, Genome Reference Consortium Human Build 37

MAF, minor allele frequency

MGRB, Medical Genome Reference Bank

N, number

N/A, not available

OCA Oculocutaneous albinism

PM pigmented melanoma

WES whole exome sequencing
