# Supplementary material for: Germline and somatic albinism variants in amelanotic/hypomelanotic melanoma: Increased carriage of TYR and OCA2 variants
Source: PLoS One. 2020 Sep 23;15(9):e0238529. doi: 10.1371/journal.pone.0238529 (PMC7510969; doi:10.1371/journal.pone.0238529)
Supplement: S2 Table — (DOCX) [file pone.0238529.s003.docx]

**Table S2: OCA3, OCA4, OCA6, OCA7 gene alleles in pigmented melanoma and amelanotic/hypomelanotic melanoma patients**

|  |  |  |  | **Controls** | | **Melanoma Cases** | | | ***X^2^* Statistical Tests (*P*-value)^l^** | | |
| --- | --- | --- | --- | --- | --- | --- | --- | --- | --- | --- | --- |
| **Gene/OCA** | **Chr:Position**  **(GRCh37)** | **HGVS Transcript Variant** | **gnomAD ^a^**  **(MAF%)** | **MGRB ^b^**  **Control**  **N**  **(MAF%)**  **Total=1144**  **WES** | **BNMS ^c^**  **Control**  **N (MAF%)**  **Total=652 ^d^** | **PM**  **N (MAF%)**  **Total=389 ^d^**  **(WES=303)** | **AHM**  **N (MAF%)**  **Total =45**  **(WES=28)^a^** | **Total Melanoma**  **Cases**  **N**  **(MAF%)**  **Total=581**  **(WES=383)** | **Total Melanoma**  **Cases vs MGRB + BNMS Control** | **AHM vs PM Cases** | **AHM Case vs MCRB + BNMS Controls** |
| ***TYRP1/*OCA3** |  |  |  |  |  |  |  |  |  |  |  |
| rs61758405*G/A  p.A24T ^e,f^ | 9:12694066 | NM_000550.2:c.70G>A | 0.3168 | 9 (0.39) | 2 (0.15) ^d^ | 3 (0.39) ^d^ | 0 (0.0) ^d^ | 4 (0.34) ^d^ | 0.741 | 0.298 | 0.476 |
| rs1481587693*G/C p.G52A | 9:12694151 | NM_000550.2:c.155G>C | 0.001^q^ | 1 (0.043) | - | 0 (0.0) | 0 (0.0) | 0 (0.0) | 0.357 | 0.511 | 0.364 |
| [rs61752864*G/A](http://www.ncbi.nlm.nih.gov/projects/SNP/snp_ref.cgi?rs=rs61752864)  p.A70T | 9:12694204 | NM_000550.2:c.208G>A | 0.04445 | 1 (0.043) | - | 0 (0.0) | 0 (0.0) | 0 (0.0) | 0.465 | 0.769 | 0.308 |
| rs61752937*G/A  p.R93H | 9:12694274 | NM_000550.2:c.278G>A | *1.112* | 10 (0.44) | 12 (0.92) ^d^ | 5 (0.64) ^d^ | 0 (0.0) ^d^ | 7 (0.60) ^d^ | 0.690 | 0.444 | 0.588 |
| rs1431631391*T/A p.C99S | 9:12694291 | NM_000550.2:c.295T>A | 0.0 | 1 (0.043) | - | 0 (0.0) | 0 (0.0) | 0 (0.0) | 0.357 | 0.513 | 0.377 |
| [rs146027807*C/T](http://www.ncbi.nlm.nih.gov/projects/SNP/snp_ref.cgi?rs=rs146027807) p.R153C ^f^ | 9:12695586 | NM_000550.2:c.457C>T | 0.07912 | 2 (0.087) | - | 0 (0.0) | 0 (0.0) | 0 (0.0) | 0.400 | 0.714 | 0.377 |
| Ch9_12695598*C/T p.P157S ^e^ | 9:12695598 | NM_000550.2:c.469C>T | 0.0 | 0 (0) | - | 1 (0.17) | 0 (0.0) | 1 (0.13) | 0.056 | 0.075 | 0.769 |
| [rs760195376*C/T](http://www.ncbi.nlm.nih.gov/projects/SNP/snp_ref.cgi?rs=rs760195376) p.R225S | 9:12695804 | NM_000550.2:c.675G>A | 0.002361 | 1 (0.043) | - | 0 (0.0) | 0 (0.0) | 0 (0.0) | 0.476 | 0.556 | 0.385 |
| [rs748170782](http://www.ncbi.nlm.nih.gov/projects/SNP/snp_ref.cgi?rs=rs748170782)*A/T p.M266L | 9:12698538 | NM_000550.2:c.796A>T | 0.0008809 | 1 (0.043) | - | 0 (0.0) | 0 (0.0) | 0 (0.0) | 0.540 | 0.645 | 0.385 |
| [rs770141693*C/G](http://www.ncbi.nlm.nih.gov/projects/SNP/snp_ref.cgi?rs=rs759390349) p.L302V | 9:12698646 | NM_000550.2:c.904C>G | 0.0 | 1 (0.043) | - | 00 (0.0) | 0 (0.0) | 0 (0.0) | 0.444 | 0.606 | 0.250 |
| rs151091299*C/G p.S305R ^e^ | 9:12702272 | NM_000550.2:c.915C>G | 0.007778 | 0 (0) | 0 (0.0) ^d^ | 1 (0.13) ^d^ | 0 (0.0) ^d^ | 1 (0.09) ^d^ | 0.051 | **0.046** | 0.513 |
| [rs377679582*C/G](http://www.ncbi.nlm.nih.gov/projects/SNP/snp_ref.cgi?rs=rs377679582) p.P346R | 9:12702394 | NM_000550.2:c.1037C>G | 0.01087 | 1 (0.043) | - | 00 (0.0) | 0 (0.0) | 0 (0.0) | 0.476 | 0.556 | 0.408 |
| rs371392847*G/A p.R502H ^e^ | 9:12709073 | NM_000550.2:c.1505G>A | 0.002650 | 0 (0) | 0 (0.0) ^d^ | 1 (0.13) ^d^ | 0 (0.0) ^d^ | 1 (0.09) ^d^ | **0.029** | 0.082 | 0.741 |
| rs770420872*A/ATGTT | 9:12708089 | NM_000550.2:c.1354del | 0.0 | 1 (0.043) | - | 00 (0.0) | 0 (0.0) | 0 (0.0) | 0.454 | 0.62 | 0.377 |
| **Allelic spectrum for *TYRP1* in each subgroup** |  |  | 10 of 14 | 11 of 14 | 2 of 4 | 5 of 14 | 0 of 14 | 5 of 14 |  |  |  |
| **Allelic spectrum for *TYRP1* (MAF<1%) in each subgroup ^g^** |  |  | 9 of 13 | 10 of 13 | 1 of 3 | 4 of 13 | 0 of 13 | 4 of 13 |  |  |  |
| **Total number of *TYRP1* alleles (combined MAF<1%) observed in each subgroup ^g^** |  |  |  | 19 (0.83) | 2 (0.15) | 6 (0.99) | 0 (0.0) | 7 (0.91) | 1.00 | 1.00 | 1.00 |
| ***SLC45A2/*OCA4** |  |  |  |  |  |  |  |  |  |  |  |
| rs775387808*G/-  p.G88fs ^e^ | 5:33984424 | NM_001297417.2:c.264del | 0.01 | 2 (0.087) | - | 1 (0.17) | 0 (0.0) | 2 (0.265) | 0.425 | 0.392 | 0.833 |
| rs150033963*C/T p.V150I ^e^ | 5: 33982455 | NM_001297417.2:c.448G>A | 0.004397 | 0 (0) | - | 1 (0.165) | 0 (0.0) | 1 (0.1325) | **0.038** | **0.042** | 0.645 |
| rs1222481616*A/G p.F158S | 5:33982430 | NM_001012509.2:c.473T>C | 0.0 | 1 (0.043) | - | 0 (0.0) | 0 (0.0) | 0 (0.0) | 0.513 | 0.513 | 0.377 |
| rs753041550*T/TA p.Y268fs | 5:33963881 | NM_016180.3:c.802dup | 0.004397 | 1 (0.043) | - | 0 (0.0) | 0 (0.0) | 0 (0.0) | 0.500 | 0.645 | 0.370 |
| [rs772902343*C/T](http://www.ncbi.nlm.nih.gov/projects/SNP/snp_ref.cgi?rs=rs772902343) p.G269D ^f^ | 5:33963878 | NM_001012509.2:c.806G>A | 0.001759 | 1 (0.043) | - | 0 (0.0) | 0 (0.0) | 0 (0.0) | 0.385 | 0.667 | 0.328 |
| rs26722*G/A  p.E272K ^i^ | 5:33963870 | NM_016180.4:c.814G>A | *1.98* | 25 **(***1.09***)** | 31 (*2.38*) ^d^ | 8 (*1.05*) ^d^ | 0 (0.0) ^d^ | 12 (*1.03*) ^d^ | 0.556 | 0.308 | 0.093 |
| [rs794727645](http://www.ncbi.nlm.nih.gov/projects/SNP/snp_ref.cgi?rs=rs794727645)*C/A p.Q344H ^f^ | 5:33954466 | NM_016180.3:c.1032G>T | 0.002641 | 1 (0.043) | - | 0 (0.0) | 0 (0.0) | 0 (0.0) | 0.465 | 0.526 | 0.339 |
| [rs760972161](http://www.ncbi.nlm.nih.gov/projects/SNP/snp_ref.cgi?rs=rs760972161)*A/T p.F360I | 5:33951737 | NM_016180.3:c.1078T>A | 0.001758 | 1 (0.043) | - | 0 (0.0) | 0 (0.0) | 0 (0.0) | 0.488 | 0.667 | 0.345 |
| rs16891982*G/C  p.L374F ^j^ | 5:33951693 | NM_016180.4:c.1122C= | *95.64* | 65 (*97.16*) | - | 589 (*97.84*) | 28 (*100*) | 1162 (*98.1*) | **0.016** | 0.278 | **0.046** |
| [rs142175557](http://www.ncbi.nlm.nih.gov/projects/SNP/snp_ref.cgi?rs=rs142175557)*G/A p.R451C | 5:33947285 | NM_016180.4:c.1351C>T | 0.008514 | 1 (0.043) | - | 0 (0.0) | 0 (0.0) | 0 (0.0) | 0.377 | 0.645 | 0.318 |
| rs142680641*G/A p.R451H ^e^ | 5:33947284 | NM_016180.4:c.1352G>A | 0.09598 | 2 (0.087) | 0 (0.0) ^d^ | 1 (0.165) ^d^ | 0 (0.0) ^d^ | 2 (0.175) ^d^ | 0.351 | 0.244 | 0.606 |
| rs779983065*C/T p.G491R ^e,f^ | 5:33944875 | NM_016180.4:c.1471G>A | 0.003 | 0 (0) | - | 1 (0.17) | 0 (0.0) | 1 (0.1325) | **0.044** | 0.089 | 0.833 |
| chr5:33944863*T/C p.G495S | 5:33944863 | NM_016180.4:c.1483G>A | 0.0 | 1 (0.043) | - | 00 (0.0) | 0 (0.0) | 0 (0.0) | 0.476 | 0.488 | 0.444 |
| [rs3733808](http://www.ncbi.nlm.nih.gov/projects/SNP/snp_ref.cgi?rs=rs3733808)*C/G  p.V507L ^g,h^ | 5:33944827 | NM_016180.3:c.1519G>C | 0.06973 | 1 (0.043) | 0 (0.0) ^d^ | 0 (0.0) ^d^ | 0 (0.0) ^d^ | 0 (0.0) ^d^ | 0.333 | 0.465 | 0.357 |
| [rs375214909*](http://www.ncbi.nlm.nih.gov/projects/SNP/snp_ref.cgi?rs=rs375214909)C/A p.V508L | 5:33944824 | NM_016180.4:c.1522G>T | 0.01162 | 3 (0.13) | - | 00 (0.0) | 0 (0.0) | 0 (0.0) | 0.298 | 0.833 | 0.345 |
| rs750510834*A/G p.C519R ^e^ | 5: 33944791 | NM_016180.4:c.1555T>C | 0.003519 | 0 (0) | - | 0 (0.0) | 0 (0.0) | 1 (0.1325) | 0.065 | 0.625 | 0.870 |
| **Allelic spectrum for *SLC45A2* in each subgroup** |  |  | 14 of 16 | 13 of 16 | 1 of 3 | 6 of 16 | 1 of 16 | 7 of 16 |  |  |  |
| **Allelic spectrum for *SLC45A2* (MAF<1%) in each subgroup ^g^** |  |  | 12 of 14 | 11 of 14 | 0 of 2 | 4 of 14 | 0 of 14 | 5 of 14 |  |  |  |
| **Total number of *SLC45A2* alleles (combined MAF<1%) observed in each subgroup ^g^** |  |  |  | 15 (0.66) | 0 (0.0) | 4 (0.66) | 0 (0) | 7 (0.91) | 1.00 | 1.00 | 1.00 |
| ***SLC24A5/*OCA6** |  |  |  |  |  |  |  |  |  |  |  |
| r[s1006909458*G/A](http://www.ncbi.nlm.nih.gov/projects/SNP/snp_ref.cgi?rs=rs1006909458) p.E56K | 15:48414098 | NM_205850.2:c.166G>A | 0.0008803 | 1 (0.043) | - | 0 (0.0) | 0 (0.0) | 0 (0.0) | 0.513 | 0.588 | 0.290 |
| [rs760875527](http://www.ncbi.nlm.nih.gov/projects/SNP/snp_ref.cgi?rs=rs760875527)*G/A p.R64H | 15:48414123 | NM_205850.2:c.191G>A | 0.0 | 1 (0.043) | - | 0 (0.0) | 0 (0.0) | 0 (0.0) | 0.444 | 0.714 | 0.400 |
| [rs142056637*](http://www.ncbi.nlm.nih.gov/projects/SNP/snp_ref.cgi?rs=rs142056637)T/G  p.Y72* ^f^ | 15:48414148 | NM_205850.2:c.216T>G | 0.003872 | 1 (0.043) | 1 (0.08) ^d^ | 0 (0.0) ^d^ | 0 (0.0) ^d^ | 0 (0.0) ^d^ | 0.625 | 0.513 | 0.444 |
| [rs1234686744*G/A](http://www.ncbi.nlm.nih.gov/projects/SNP/snp_ref.cgi?rs=rs1234686744) p.M79I | 15:48414169 | NM_205850.2:c.237G>A | 0.0008795 | 1 (0.043) | - | 0 (0.0) | 0 (0.0) | 0 (0.0) | 0.454 | 0.500 | 0.333 |
| rs1426654*G/A  p.A111T ^k^ | 15:48426484 | NM_205850.2:c.331A>G | *99.63* | 2,277 (*99.52*) | 1,276 (*97.85*) ^d^ | 762  (*99.74*) ^d^ | 90 (*100*) ^d^ | 1060 (*99.74*) ^d^ | 0.465 | 0.769 | 0.374 |
| chr15:48429046*T/G p.E253* | 15:48429046 | NM_205850.2:c.757G>T | 0.0 | 1 (0.043) | - | 0 (0.0) | 0 (0.0) | 0 (0.0) | 0.465 | 0.606 | 0.328 |
| [rs762506377*C/-](http://www.ncbi.nlm.nih.gov/projects/SNP/snp_ref.cgi?rs=rs762506377) p.P299fs | 15:48431188 | NM_205850.2:c.896del | 0.001555 | 1 (0.043) | - | 0 (0.0) | 0 (0.0) | 0 (0.0) | 0.488 | 0.556 | 0.303 |
| [rs753732275*G/A](http://www.ncbi.nlm.nih.gov/projects/SNP/snp_ref.cgi?rs=rs753732275) p.W330* ^f^ | 15:48431283 | NM_205850.2:c.989G>A | 0.02258 | 1 (0.043) | - | 0 (0.0) | 0 (0.0) | 0 (0.0) | 0.385 | 0.4878 | 0.417 |
| [rs147788058*C/A](http://www.ncbi.nlm.nih.gov/projects/SNP/snp_ref.cgi?rs=rs147788058) p.P366T ^f^ | 15:48433327 | NM_205850.2:c.1096C>A | 0.01785 | 1 (0.043) | - | 0 (0.0) | 0 (0.0) | 0 (0.0) | 0.488 | 0.588 | 0.444 |
| [rs775030415*G/A](http://www.ncbi.nlm.nih.gov/projects/SNP/snp_ref.cgi?rs=rs775030415) p.D367N | 15:48433330 | NM_205850.2:c.1099G>A | 0.003526 | 1 (0.043) | - | 0 (0.0) | 0 (0.0) | 0 (0.0) | 0.385 | 0.571 | 0.345 |
| chr15:48434252*A/C p.I403L | 15:48434252 | NM_205850.2:c.1207A>C | 0.0 | 1 (0.043) | - | 0 (0.0) | 0 (0.0) | 0 (0.0) | 0.400 | 0.588 | 0.339 |
| [rs372932575*T/A](http://www.ncbi.nlm.nih.gov/projects/SNP/snp_ref.cgi?rs=rs372932575) p.L454* ^f^ | 15:48434406 | NM_205850.2:c.1361T>A | 0.007925 | 1 (0.043) | - | 0 (0.0) | 0 (0.0) | 0 (0.0) | 0.435 | 0.645 | 0.377 |
| **Allelic spectrum for *SLC24A5* in each subgroup** |  |  | 9 of 12 | 12 of 12 | 2 of 2 | 1 of 12 | 1 of 12 | 1 of 12 |  |  |  |
| **Allelic spectrum for *SLC24A5* (MAF<1%) in each subgroup ^g^** |  |  | 8 of 11 | 11 of 11 | 1 of 1 | 0 of 11 | 0 of 11 | 0 of 11 |  |  |  |
| **Total number of *SLC24A5* alleles (combined MAF<1%) observed in each subgroup ^g^** |  |  |  | 11 (0.48) | 1 (0.07) | 0 (0.0) | 0 (0.0) | 0 (0.0) | **0.02** | 1.00 | 1.00 |
| ***LRMDA/*OCA7** |  |  |  |  |  |  |  |  |  |  |  |
| rs146123023*TC/T p.S8AfsTer14 | 10:77542753 | NM_032024.3:c.21del | 0.1358 | 3 (0.13) | - | 0 (0.0) | 0 (0.0) | 0 (0.0) | 0.312 | 0.741 | 0.253 |
| rs757561647*G/T p.A86S | 10:77807003 | NM_032024.4:c.256G>T | 0.00088 | 1 (0.043) | - | 0 (0.0) | 0 (0.0) | 0 (0.0) | 0.540 | 0.645 | 0.465 |
| rs200848450*C/T p.R128* | 10:77818491 | NM_032024.3:c.382C>T | 0.0008791 | 0 (0) | 1 (0.08) ^d^ | 0 (0.0) ^d^ | 0 (0.0) ^d^ | 0 (0.0) ^d^ | 0.667 | 0.500 | 0.312 |
| rs35349706*C/T  p.S153F ^b^ | 10:78084184 | NM_032024.4:c.458C>T | *1.3* | 23 (*1.01*) | 20 (*1.53*) | 8 (*1.05*) | 2 (***2.2***) | 13 (*1.12*) | 0.200 | 0.500 | 0.640 |
| rs75852090*G/A p.G176E | 10: 78316976 | NM_032024.3:c.527G>A | 0.02168 | 0 (0) | 0 (0.0) | 0 (0.0) | 0 (0.0) | 1 (0.09) | 0.055 | 0.556 | 0.741 |
| **Allelic spectrum for *LRMDA* in each subgroup** |  |  | 5 of 5 | 3 of 5 | 2 of 3 | 2 of 5 | 1 of 5 | 2 of 5 |  |  |  |
| **Allelic spectrum for *LRMDA*(MAF<1%) in each subgroup ^g^** |  |  | 4 of 4 | 2 of 4 | 1 of 2 | 0 of 4 | 0 of 4 | 1 of 4 |  |  |  |
| **Total number of *LRMDA* alleles (combined MAF<1%) observed in each subgroup ^g^** |  |  |  | 5 (0.22) | 1 (0.08) | 0 (0.0) | 0 (0.0) | 1 (0.09) | 0.67 | 1.00 | 1.00 |

^a^ Lek et al., 2016 [39] <http://exac.broadinstitute.org>. European Non-Finnish, listing the minor allele frequency.

^b^ Lacaze et al., 2019 [33], The Medical Genome Reference Bank

^c^ Duffy et al., 2020 [22]

^d^ Illumina Core Exome genotyping

^e^ Deleterious by in silico analysis using Polyphen2 [30] or MutationTaster [32], with all prediction tools shown in Supplementary File 5

^f^ Pathogenic Albinism variant reported by Lasseaux et al., 2018 [3]

^g^ Albinism database <http://www.ifpcs.org/albinism/oca1mut.html>, albinism allele

^h^ Albinism database <http://www.ifpcs.org/albinism/oca1mut.html>, polymorphism

^i^ p.E272K skin darkening [6], gnomAD East Asian frequency = 39.71%

^j^ p.L374F skin lightening [6], gnomAD East Asian frequency = 0.19%

^k^ p.A111T skin lightening [7], gnomAD East Asian frequency = 0.69%

^l^ Bonferroni corrected (three phenotypes x 33 variants) critical *P=*0.0005 equivalent to a table wide α=0.05

AHM, amelanotic/hypomelanotic melanoma

BNMS, Brisbane Naevus Morphology Study

GRCh37, Genome Reference Consortium Human Build 37

MAF, minor allele frequency

MGRB, Medical Genome Reference Bank

N, number

OCA, Oculocutaneous albinism

PM, pigmented melanoma

WES, whole exome sequencing
