# Supplementary material for: Germline and somatic albinism variants in amelanotic/hypomelanotic melanoma: Increased carriage of TYR and OCA2 variants
Source: PLoS One. 2020 Sep 23;15(9):e0238529. doi: 10.1371/journal.pone.0238529 (PMC7510969; doi:10.1371/journal.pone.0238529)
Supplement: S3 Table — (DOCX) [file pone.0238529.s004.docx]

**Table S3: Pigmentation gene alleles in pigmented melanoma and amelanotic/hypomelanotic melanoma patients**

|  |  |  |  | **Controls** | | **Melanoma Cases** | | | ***X^2^* Statistical Tests (*P*-value) ^f^** | | |
| --- | --- | --- | --- | --- | --- | --- | --- | --- | --- | --- | --- |
| **Pigmentation Gene** | **Chr:Position**  **(GRCh37)** | **HGVS Transcript Variant** | **gnomAD**  **(MAF%)** **^a^** | **MGRB ^b^**  **Control**  **N (MAF%)**  **Total=1144**  **WES** | **BNMS ^c^**  **Controls**  **N (MAF %)**  **Total=652 ^d^** | **PM**  **N (MAF%)**  **Total=389 ^d^**  **(WES=303)** | **AHM**  **N (MAF%)**  **Total=45 ^d^**  **(WES=28)** | **Total Melanoma**  **Cases**  **N (MAF%)**  **Total=581 ^d^**  **(WES=283)** | **Total Melanoma**  **Cases vs MGRB + BNMS Controls** | **AHM vs PM Cases** | **AHM Case vs MGRB + BNMS Controls** |
| ***KITLG*** |  |  |  |  |  |  |  |  |  |  |  |
| rs41283112*C/A p.D210Y | 12:88900891 | NM_000899.4:c.628G>T | *1.6* | 32/1144 (1.4%) | 21 (*1.6*) ^d^ | 11 (*1.44*) ^d^ | 3 (*3.33*) ^d^ | 25 (*2.15***)** ^d^ | 0.267 | 0.227 | 0.298 |
| rs3741457*T/C  p.T54A | 12:88926250 | NM_000899.4:c.160A>G | 0.003114 | 0 (0.0) | 1 (0.077)  ^d^ | 0 (0.0)  ^d^ | 0 (0.0) ^d^ | 0 (0.0) ^d^ | 0.454 | 0.571 | 0.345 |
| rs12821256*T/C | 12:89328335 | NC_000012.11:g.89328335T>C | *11.07* | 254 (*11.10*) | 153 (*11.73*)  ^d^ | 74 (*9.71*) ^d^ | 8 (*9.09*) ^d^ | 111 (*9.55*) ^d^ | 0.189 | 0.650 | 0.870 |
| ***POMC*** |  |  |  |  |  |  |  |  |  |  |  |
| rs143923583*T/C p.R25H | 2:25387568 | NM_001319204.1:c.74G>A | 0.002323 | 1 (0.04) | - | 0 (0.0) | 0 (0.0) | 0 (0.0) | 0.526 | 0.667 | 0.364 |
| rs1427018985*C/T p.D66N | 2:25384558 | NM_001319205.1:c.196G>A | 0.006186 | 1 (0.04) | - | 0 (0.0) | 0 (0.0) | 0 (0.0) | 0.500 | 0.667 | 0.377 |
| rs201517327*T/C p.N91S | 2:25384482 | NM_001319204.1:c.272A>G | 0 | 0 (0.0) | 1 (0.08) ^d^ | 0 (0.0) ^d^ | 0 (0.0) ^d^ | 0 (0.0) ^d^ | 0.308 | 0.500 | 0.465 |
| rs201519174*G/C p.H143Q **^e^** | 2:25384325 | NM_001319205.1:c.429C>G | 0.033 | 2 (0.087) | - | 0 (0.0) | 1 (1.79) | 1 (0.09) | 0.6897 | **0.014** | **0.028** |
| rs201408477*A/G p.F144L | 2:25384324 | NM_000939.2:c.430T>C | 0.02869 | 1 (0.04) | - | 0 (0.0) | 0 (0.0) | 0 (0.0) | 0.3390 | 0.741 | 0.392 |
| rs80326661*T/C p.E214G | 2:25384113 | NM_000939.2:c.641A>G | 0.9313 | 14 (0.619) | - | 2 (0.33) | 0 (0.0) | 2 (0.18) | 0.1550 | 0.690 | 0.339 |
| rs149540566*T/C p.Y221C **^e^** | 2:25384092 | NM_001319205.1:c.662A>G | 0.1554 | 6 (0.262) | 2 (0.15) ^d^ | 5 (0.72) ^d^ | 0 (0.0) ^d^ | 5 (0.43) ^d^ | 0.179 | 0.103 | 0.526 |
| rs1423739145*G/T p.F226L | 2:25384076 | NM_001035256.2:c.678C>A | 0 | 1 (0.04) | - | 0 (0.0) | 0 (0.0) | 0 (0.0) | 0.454 | 0.571 | 0.317 |
| rs28932472*G/C p.R236G  **^e^** | 2:25384048 | NM_000939.2:c.706C>G | 0.39 | 17 (0.743) | - | 4 (0.66) | 1 (1.79) | 5 (0.43) | 0.952 | 0.741 | 0.800 |
| ***SLC24A4*** |  |  |  |  |  |  |  |  |  |  |  |
| rs12896399*G/T | 14:92773663 | NM_153646.3:c.232A>G | *44.23* | 988 (*43.18*) | 569 (*43.63*) ^d^ | 339 (*44.49*) ^d^ | 47 (*53.41*) ^d^ | 531 (*45.7*) ^d^ | 0.227 | 0.180 | 0.190 |
| rs150573991*A/G p.T78A | 14:92792313 | NM_153647.3:c.242C>T | 0.07 | 1 (0.04) | 2 (0.15) ^d^ | 1 (0.17) ^d^ | 0 (0.0) ^d^ | 1 (0.09) ^d^ | 0.952 | 0.333 | 0.385 |
| rs139559738*C/T p.A81V | 14:92900256 | NM_153646.3:c.408C>T | 0.02942 | 1 (0.04) | - | 0 (0.0) | 0 (0.0) | 0 (0.0) | 0.526 | 0.588 | 0.408 |
| rs139530271*C/T p.S136S | 14:92908447 | NM_153646.3:c.412G>C | 0.00077 | 1 (0.04) | - | 0 (0.0) | 0 (0.0) | 0 (0.0) | 0.500 | 0.571 | 0.377 |
| rs771759475*G/C p.D138H | 14:92908451 | NM_153647.3:c.473T>C | 0.001? | 1 (0.04) | - | 0 (0.0) | 0 (0.0) | 0 (0.0) | 0.556 | 0.833 | 0.435 |
| rs75348968*T/C p.V158A | 14:92908512 | NM_153647.3:c.520G>A | 0.1008 | 3 (0.13) | - | 1 (0.17) | 0 (0.0) | 1 (0.09) | 0.870 | 0.385 | 0.465 |
| rs774866159*G/A p.V174M | 14:92909098 | NM_153646.3:c.565G>A | 0.004 | 0 (0.0) | - | 1 (0.17) | 0 (0.0) | 1 (0.09) | 0.060 | 0.174 | 0.714 |
| rs140536494*G/A p.G189R | 14:92909143 | NM_153646.3:c.800C>T | 0.01008 | 1 (0.04) | 4 (0.31) ^d^ | 0 (0.0) ^d^ | 0 (0.0) ^d^ | 0 (0.0) ^d^ | 0.103 | 0.870 | 0.163 |
| rs148253052*C/T p.P267L | 14:92915480 | NM_153646.3:c.899A>G | 0.00264 | 1 (0.04) | - | 0 (0.0) | 0 (0.0) | 0 (0.0) | 0.377 | 0.625 | 0.312 |
| rs139572787*A/G p.Y300C | 14:92920262 | NM_153646.3:c.920T>C | 0.15 | 3 (0.13) | 4 (0.31) ^d^ | 1 (0.13) ^d^ | 0 (0.0) ^d^ | 1 (0.09) ^d^ | 0.571 | 0.556 | 0.290 |
| rs1423708858*T/C p.M307T | 14:92920283 | NM_153646.3:c.920T>C | 0.00088 | 1 (0.04) | - | 0 (0.0) | 0 (0.0) | 0 (0.0) | 0.526 | 0.540 | 0.357 |
| rs751411026*A/G p.M312I | 14:92920299 | NM_153646.3:c.936G>A | 0.001761 | 0 (0.0) | - | 1 (0.13) | 0 (0.0) | 1 (0.09) | 0.071 | 0.078 | 0.833 |
| rs200923561*C/T p.R351Y | 14:92922748 | NM_153646.3:c.1051C>T | 0 | 0 (0.0) | 1 (0.077) ^d^ | 0 (0.0) ^d^ | 0 (0.0) ^d^ | 0 (0.0) ^d^ | 0.377 | 0.513 | 0.425 |
| rs751462597*G/A p.R351Q | 14:92922749 | NM_153646.3:c.1052G>A | 0.00615 | 1 (0.04) | - | 0 (0.0) | 0 (0.0) | 0 (0.0) | 0.465 | 0.540 | 0.312 |
| rs199580422*A/T p.I488F | 14:92953049 | NM_153646.3:c.1462A>T | 0.01 | 0 (0.0) | - | 1 (0.17) | 0 (0.0) | 1 (0.09) | **0.040** | 0.096 | 0.667 |
| rs142889151*C/T p.A510V | 14:92953116 | NM_153646.3:c.1529C>T | 0.114 | 3 (0.13) | 1 (0.08) ^d^ | 0 (0.0) ^d^ | 0 (0.0) ^d^ | 0 (0.0) ^d^ | 0.180 | 0.690 | 0.500 |
| rs200401376*T/C p.M543T | 14:92958099 | NM_153646.3:c.1628T>C | 0.00543 | 1 (0.04) | - | 0 (0.0) | 0 (0.0) | 0 (0.0) | 0.317 | 0.690 | 0.444 |
| rs45587635*C/A p.K552Q | 14:92958522 | NM_153646.3:c.1654A>C | ***4.707*** | 115 (*5.03*) | 60 (*4.54*) ^d^ | 35 (*4.58*) ^d^ | 2 (*2.22*) ^d^ | 51 (*4.45*) ^d^ | 0.800 | 0.298 | 0.185 |
| chr14:92959833*G/A p.H577R | 14:92959833 | NM_153646.3:c.1730A>G | - | 1 (0.04) | - | 0 (0.0) | 0 (0.0) | 0 (0.0) | 0.425 | 0.625 | 0.408 |
| chr14:92959834*T/C p.H577H | 14:92959834 | NM_153646.3:c.1731C>T | - | 1 (0.04) | - | 0 (0.0) | 0 (0.0) | 0 (0.0) | 0.645 | 0.513 | 0.317 |
| rs4900130*G/A p.V613I | 14:92959940 | NM_153646.3:c.1837G>A | 1.1 | 16 (0.70) | 16 (*1.23*) ^d^ | 5 (0.65) ^d^ | 2 (*2.22*) ^d^ | 12 (*1.03*) ^d^ | 0.339 | 0.278 | 0.571 |
| ***TPCN2*** |  |  |  |  |  |  |  |  |  |  |  |
| rs78455795*C/T p.R84W | 11:68822264 | NM_139075.3:c.250C>T | 0.006980 | 0 (0.0) | 4 (0.31) ^d^ | 0 (0.0) ^d^ | 0 (0.0) ^d^ | 0 (0.0) ^d^ | 0.127 | 0.667 | 0.192 |
| rs79490424*A/G p.V163M | 11:68825103 | NM_139075.3:c.487G>A | 0.1163 | 2 (0.087) | 0 (0.0) ^d^ | 0 (0.0) ^d^ | 0 (0.0) ^d^ | 0 (0.0) ^d^ | 0.476 | 0.833 | 0.645 |
| rs200456521*C/T p.L184P | 11:68830356 | NM_139075.3:c.551T>C | 0.003519 | 1 (0.04) | - | 0 (0.0) | 0 (0.0) | 0 (0.0) | 0.345 | 0.714 | 0.392 |
| rs139760549*C/T p.P214L | 11:68830446 | NM_139075.3:c.641C>T | 0.0039 | 0 (0.0) | - | 0 (0.0) | 0 (0.0) | 1 (0.09) | **0.045** | 0.588 | 0.741 |
| rs72928978*G/A p.V219I | 11-68831364 | NM_139075.3:c.655G>A | *12.98* | 247 (*10.79*) | 132 (*10.12*) ^d^ | 82 (*10.73*) ^d^ | 8 (*8.88*) ^d^ | 119 (*10.24*) ^d^ | 0.833 | 0.741 | 0.690 |
| rs374995591*T/G p.A224S | 11:68831379 | NM_139075.3:c.670G>T | 0.01717 | 1 (0.04) | - | 0 (0.0) | 0 (0.0) | 0 (0.0) | 0.526 | 0.714 | 0.256 |
| rs142288453*G/A p.A272T | 11:68835058 | NM_139075.3:c.814G>A | 0.08083 | 1 (0.04) | 0 (0.0) ^d^ | 0 (0.0) ^d^ | 0 (0.0) ^d^ | 0 (0.0) ^d^ | 0.690 | 0.625 | 0.444 |
| rs150335100*T/G p.G317V | 11:68838878 | NM_139075.3:c.950G>T | 0.02257 | 1 (0.04) | 0 (0.0 ) ^d^ | 0 (0.0) ^d^ | 0 (0.0) ^d^ | 0 (0.0) ^d^ | 0.4878 | 0.540 | 0.247 |
| rs3750965*A/G p.K376R | 11-68840160 | NM_139075.3:c.1127A>G | *30.88* | 624 (*27.27*) | 357 (*27.38*) ^d^ | 222 (*29.06*) ^d^ | 25 (*27.78*) ^d^ | 318 (*27.37*) ^d^ | 0.333 | 0.282 | 0.357 |
| rs35264875*A/T p.M484L | 11:68846399 | NM_139075.3:c.1450A>T | *18.52* | 343 (*14.99*) | 88 (*13.02*) ^d^ | 105 (*15.35*) ^d^ | 11 (*16.18*) ^d^ | 152 (*15.51*) ^d^ | 0.435 | 0.741 | 0.714 |
| rs145186935*C/T p.R524* | 11:68847332 | NM_139075.3:c.1570C>T | 0.006205 | 2 (0.087) | - | 0 (0.0) | 0 (0.0) | 0 (0.0) | 0.294 | 0.714 | 0.270 |
| rs1447898591*C/T p.S539L | 11:68848894 | NM_139075.3:c.1616C>T | 0.002729 | 1 (0.04) | - | 0 (0.0) | 0 (0.0) | 0 (0.0) | 0.323 | 0.769 | 0.351 |
| rs34510004*G/A p.M546I | 11:68848916 | NM_139075.3:c.1638G>A | 0.112 | 3 (0.13) | 0 (0.0) ^d^ | 2 (0.26) ^d^ | 0 (0.0) ^d^ | 3 (0.26) ^d^ | 0.351 | 0.187 | 0.606 |
| rs148079137*G/A p.R554H | 11:68848939 | NM_139075.3:c.1661G>A | 0.02925 | 1 (0.04) | - | 0 (0.0) | 0 (0.0) | 0 (0.0) | 0.454 | 0.571 | 0.270 |
| rs2376558* C/T p.P564L | 11:68851414 | NM_139075.3:c.1691T>C | 0.95 | 9 (0.39) | 19 (1.46)  ^d^ | 2 (0.26) ^d^ | 1 (1.1) ^d^ | 4 (0.34) ^d^ | 0.185 | 0.465 | 0.625 |
| rs770971452*G/A p.V568M | 11:68851425 | NM_139075.3:c.1702G>A | 0.006990 | 1 (0.04) | - | 0 (0.0) | 0 (0.0) | 0 (0.0) | 0.435 | 0.645 | 0.308 |
| rs147393231*G/A p.G583S | 11:68851470 | NM_139075.3:c.1747G>A | 0.062 | 0 (0.0) | 3 (0.23) ^d^ | 1 (0.17) ^d^ | 0 (0.0) ^d^ | 1 (0.09) ^d^ | 0.909 | 0.182 | 0.400 |
| rs144905179*G/A p.A633T | 11:68853197 | NM_139075.3:c.1897G>A | 0.0596 | 0 (0.0) | 0 (0.0) ^d^ | 0 (0.0) ^d^ | 0 (0.0) ^d^ | 1 (0.09) ^d^ | 0.067 | 0.556 | 0.870 |
| rs747682582*A/C p.M650L | 11:68853343 | NM_139075.3:c.1948A>C | 0.001761 | 1 (0.04) | - | 0 (0.0) | 0 (0.0) | 0 (0.0) | 0.500 | 0.588 | 0.377 |
| rs770687181*G/A p.R664H | 11:68853386 | NM_139075.3:c.1991G>A | 0.0044 | 0 (0.0) | - | 1 (0.17) | 0 (0.0) | 1 (0.09) | **0.049** | 0.091 | 0.833 |
| rs371515324*C/T p.P668S | 11:68853397 | NM_139075.3:c.2002C>T | 0.005 | 0 (0.0) | - | 0 (0.0) | 1 (1.79) | 1 (0.09) | 0.056 | **0.006** | **0.003** |
| rs1171396908*A/G p.Y673C | 11:68854005 | NM_139075.3:c.2018A>G | - | 0 (0.0) | - | 1 (0.17) | 0 (0.0) | 1 (0.09) | 0.073 | 0.071 | 0.690 |
| rs78034812*C/T p.S681L | 11:68854029 | NM_139075.3:c.2042C>T | 0.55 | 3 (0.13) | 4 (0.31) ^d^ | 1 (0.17) ^d^ | 0 (0.0) ^d^ | 1 (0.09) ^d^ | 0.3078 | 0.952 | 0.952 |
| rs150476703*A/G p.N687S | 11:68854047 | NM_139075.3:c.2060A>G | 0.04339 | 1 (0.04) | 0 (0.0) ^d^ | 0 (0.0) ^d^ | 0 (0.0) ^d^ | 0 (0.0) ^d^ | 0.690 | 0.571 | 0.526 |
| rs3829241*G/A p.G734E | 11:68855363 | NM_139075.3:c.2201G>A | *38.63* | 896 (*39.16*) | 552 (*42.33*) ^d^ | 301 (*39.5*) ^d^ | 35 (*39.77*) ^d^ | 467 (*40.19*) ^d^ | 0.640 | 1 | 0.960 |

^a^ Lek et al., 2016 [39] <http://exac.broadinstitute.org>. European Non-Finnish, listing the minor allele frequency.

^b^ Lacaze et al., [33] The Medical Genome Reference Bank

^c^ Duffy et al., 2020 [22]

^d^ Illumina Core Exome genotyping

^e^ Deleterious by in silico analysis using Polyphen2 [30] or MutationTaster [32], with all prediction tools shown in Supplementary File 5

^f^ Bonferroni corrected (three phenotypes x 49 variants) critical *P=*0.00003 equivalent to a table wide α=0.05

AHM amelanotic/hypomelanotic melanoma

GRCh37 Genome Reference Consortium Human Build 37

PM pigmented melanoma
