# Supplementary material for: Germline and somatic albinism variants in amelanotic/hypomelanotic melanoma: Increased carriage of TYR and OCA2 variants
Source: PLoS One. 2020 Sep 23;15(9):e0238529. doi: 10.1371/journal.pone.0238529 (PMC7510969; doi:10.1371/journal.pone.0238529)
Supplement: S4 Table — (DOCX) [file pone.0238529.s005.docx]

**Table S4. *MC1R* genotype and allele frequencies in PM and AHM from the TCGA SKCM collection**

| ***MC1R* Genotype*^a^*** | **N (%)** | **Other Cancers N (%)** | **PM*^b^***  **N (%)** | **AHM*^b^***  **N (%)** |
| --- | --- | --- | --- | --- |
| WT/WT | 3,854 (37.1) | 3,755 (37.9) | 91 (19.7) | 0 (0.0) |
| WT/R | 1,596 (15.4) | 1,494 (15.1) | 105 (22.7) | 2 (28.6) |
| WT/r | 2,725 (26.2) | 2,622 (26.4) | 101 (21.8) | 1 (14.3) |
| R/R | 260 (2.5) | 228 (2.3) | 36 (7.8) | 0 (0.0) |
| R/r | 728 (7.0) | 652 (6.6) | 73 (15.8) | 3 (42.8) |
| r/r | 1,226 (11.8) | 1,168 (11.8) | 57 (12.3) | 1 (14.3) |
| Total | 10,389 (100) | 9,919 (100) | 463 (100) | 7 (100) |
|  |  |  |  |  |
| ***MC1R* Allele*^a^*** | **N (%)** | **Other Cancers N (%)** | **PM*^b^***  **N (%)** | **AHM*^b^***  **N (%)** |
| WT | 12,016 (57.83) | 11,625 (58.6) | 388 (41.9) | 3 (21.4) |
| R | 2,854 (13.74) | 2,599 (13.1) | 250 (27.0) | 5 (35.7) |
| r | 5,908 (28.43) | 5,614 (28.3) | 288 (31.1) | 6 (42.9) |
| Total | 20,778 (100) | 19,838 (100) | 926 (100) | 14 (100) |

***^a^*** Common *MC1R* alleles designated in Ainger et al., 2017 [4]

***^b^*** Includes *MC1R* rare alleles designated in Robles-Espinoza et al., 2016 [48]
