# Supplementary material for: Germline and somatic albinism variants in amelanotic/hypomelanotic melanoma: Increased carriage of TYR and OCA2 variants
Source: PLoS One. 2020 Sep 23;15(9):e0238529. doi: 10.1371/journal.pone.0238529 (PMC7510969; doi:10.1371/journal.pone.0238529)
Supplement: Attachment — Submitted filename: Screen Shot 2020-02-26 at 5.09.15 pm.png.pdf [file pone.0238529.s011.pdf]

Exploration | Ssms | Mail - David.Duffy@qimrberghc | albinism genes registry at Duck | Albinism NGS Panel - Tests - G

https://portal.gdc.cancer.gov/exploration?cases_size=40&facetTab=genes&filters={"op"%3A"and"%2C"content"%3A[{"op"%3A"in"%2C"content"%3A{"field"%3A"cases.diagnoses.primary_diagnosis" 90%

Join us for our next webinar on GDC Clinical Data Analysis on February 24 at 2pm EST. Follow this link for more information. Dismiss

NIH NATIONAL CANCER INSTITUTE GDC Data Portal | Home | Projects | Exploration | Analysis | Repository | Quick Search | Manage Sets | Login | Cart 0 | GDC Apps

Cases | Clinical | Genes | Mutations

Gene

- SNAI2
- DTNBP1
- KITLG
- RAB27A
- TYR
- SOX10
- GPR143
- OCA2
- TYRP1
- MLPH
- EDN3
- PAX3
- SLC24A4
- LYST
- CDKN2A
- C10orf11
- ATM
- KIT
- BRAF
- SLC45A2
- MITF
- SLC24A5
- MYO5A
- NRAS

e.g. BRAF, ENSG00000157764

Upload Gene Set

Biotype

- protein_coding 14

Is Cancer Gene Census

- true 5

Clear | Primary Diagnosis IS amelanotic melanoma AND Project Id IS TCGA-SKCM AND Gene IN (SNAI2 DTNBP1 ...)

View Files in Repository

Cases (7) | Genes (14) | Mutations (14) | OncoGrid

Genes

Distribution of Most Frequently Mutated Genes

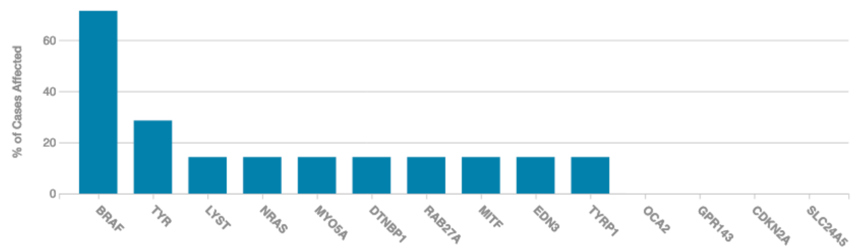

Overall Survival Plot

Not enough survival data

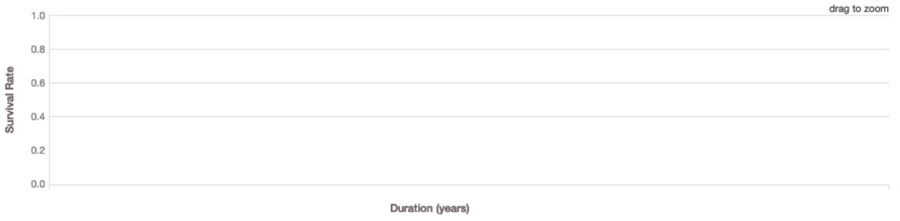

Showing **1** - **14** of **14** genes

JSON | TSV | Save/Edit Gene Set

Symbol	Name	# SSM Affected Cases in Cohort	# SSM Affected Cases Across the GDC	# CNV Gain	# CNV Loss	# Mutations	Annotations	Survival
BRAF	B-Raf proto-oncogene, serine/threonine kinase	5 / 7 (71.43%)	813 / 10,202	0 / 7 (0.00%)	0 / 7 (0.00%)	3		
TYR	tyrosinase	2 / 7 (28.57%)	226 / 10,202	0 / 7 (0.00%)	0 / 7 (0.00%)	2		
LYST	lysosomal trafficking regulator	1 / 7 (14.29%)	602 / 10,202	0 / 7 (0.00%)	0 / 7 (0.00%)	1		
NRAS	neuroblastoma RAS viral (v-ras) oncogene homolog	1 / 7 (14.29%)	323 / 10,202	0 / 7 (0.00%)	0 / 7 (0.00%)	1		
MYO5A	myosin VA (heavy chain 12, myoxin)	1 / 7 (14.29%)	319 / 10,202	0 / 7 (0.00%)	2 / 7 (28.57%)	2		
DTNBP1	dystrobrevin binding protein 1	1 / 7 (14.29%)	108 / 10,202	1 / 7 (14.29%)	0 / 7 (0.00%)	1		
RAB27A	RAB27A, member RAS oncogene family	1 / 7 (14.29%)	64 / 10,202	1 / 7 (14.29%)	1 / 7 (14.29%)	1		
MITF	microphthalmia-associated transcription factor	1 / 7 (14.29%)	198 / 10,202	1 / 7 (14.29%)	0 / 7 (0.00%)	1		
EDN3	endothelin 3	1 / 7 (14.29%)	121 / 10,202	0 / 7 (0.00%)	0 / 7 (0.00%)	1		
TYRP1	tyrosinase-related protein 1	1 / 7 (14.29%)	146 / 10,202	0 / 7 (0.00%)	0 / 7 (0.00%)	1		
OCA2	oculocutaneous albinism II	0 / 7 (0.00%)	357 / 10,202	0 / 7 (0.00%)	1 / 7 (14.29%)	0		
GPR143	G protein-coupled receptor 143	0 / 7 (0.00%)	91 / 10,202	1 / 7 (14.29%)	0 / 7 (0.00%)	0		
CDKN2A	cyclin-dependent kinase inhibitor 2A	0 / 7 (0.00%)	514 / 10,202	0 / 7 (0.00%)	4 / 7 (57.14%)	0		
SLC24A5	solute carrier family 24 (sodium/potassium/calcium exchanger), member 5	0 / 7 (0.00%)	133 / 10,202	0 / 7 (0.00%)	2 / 7 (28.57%)	0		

Show 20 entries

8 | Highlight All | Match Case | Whole Words
