# Supplementary material for: Germline and somatic albinism variants in amelanotic/hypomelanotic melanoma: Increased carriage of TYR and OCA2 variants
Source: PLoS One. 2020 Sep 23;15(9):e0238529. doi: 10.1371/journal.pone.0238529 (PMC7510969; doi:10.1371/journal.pone.0238529)
Supplement: Attachment — Submitted filename: Response to Reviewers.docx [file pone.0238529.s012.docx]

The Editors

*PLOS One*

https://journals.plos.org/plosone

Friday 26^th^ June, 2020

Dear Editors:

We are pleased to hear that you are willing to consider a revised version of our manuscript no: PONE-D-20-07313 as an **Original Article** in *PLOS One.*

**New Title:** Germline and somatic albinism variants in amelanotic/hypomelanotic melanoma: increased carriage of *TYR* and *OCA2* variants

**Authors:** Jenna E. Rayner, David L. Duffy, Darren J. Smit, Kasturee Jagirdar, Katie J. Lee, Brian De’Ambrosis, B. Mark Smithers, Erin K. McMeniman, Aideen M. McInerney-Leo, Helmut Schaider, Mitchell S. Stark, H. Peter Soyer and Richard A. Sturm

The revised manuscript has addressed the reviewers’ concerns and these changes have been added to the electronic version of “Revised Manuscript with Track Changes.docx”, and the responses described below. The text is now 4890 words, Figure 1 has been modified as have all Tables and Supplementary Tables. We apologise that the submission is being sent after 5^th^ of June suggested for preparation of the revision.

This cover note is included as an attachment “Response to Reviewers.docx”. The manuscript with highlighted changes is uploaded as “Revised Manuscript with Track Changes.docx”. The unmarked version of the manuscript is uploaded as “Revised Manuscript Clean Copy.docx”.

**Editorial Comments:**

**Please address the points brought up by the reviewers. Additionally, all the tables, including supplementary, have to clearly indicate the statistical tests used. There is lot of information provided in footnotes, but not on statistical analyses.**

This is now done, in addition to the other changes described below.

Table 1 has removed the denoted statistical tests in the footnotes and these are now in the headings of the columns.

Table 2 has been modified to spell out Odds Ratio and Trend *X^2^* (*P*-value).

Table 3 has the use of fonts corrected.

Table 4 has been modified to spell out Odds Ratio and define Pr(>Chi) in the footnotes. The significance is now defined for **, ***

Table 5 has been corrected.

Table S1, S2 and S3 now spell out *X^2^* Statistical Tests (*P*-value) in the heading to the columns and Table S1 now explicit where the SMMAT test is used in the headings.

Table S4 has removed the track change highlights and references now in correct format.

**The point of multiple testing was brought up in the previous review but not addressed. If you suggest that SMMAT takes care of this issue, it has to be explained.**

The original comment and rebuttal in our first cover letter was,

**>2.	Multiple testing correction was not mentioned either for single-variant testing or >gene-based testing.**

>Although we present X^2^ tests for each variant and report the significance in the supplementary

>Tables, the SMMAT test is more appropriate that allows for the number of variants per gene.

We have added these sentences to the Materials and methods section, under the heading Statistical analysis

“Allele frequency of each variant in each subgroup was compared using contingency *X*^2^ tests. All P-values are unadjusted for multiple testing, but should be interpreted as nested within the gene based tests.”

And two sentences below this,

“This correctly adjusts for the presence of multiple variants contributing to the test.”

In the results section we have modified these sentences,

“In considering the frequency of individual rare *TYR* variants in different subtypes of melanoma, the p.A23T variant showed the largest difference, occurring at a higher frequency in AHM vs PM (*X*^2^ unadjusted *P*=0.008).”

“The p.A23T and p.T373K variants were more common in AHM cases as compared to controls (*X*^2^ unadjusted *P*=0.006 and *X*^2^ *P*=0.02 respectively).”

“However, analyzing each variant separately revealed that the p.V443I variant occurred at higher frequency in AHM cases (4.44%) compared to PM cases (1.57%), BNMS controls (0.84%), or the MGRB (1.09%) (combined control comparison *X*^2^ *P*=.01), with comparison of any case nominally significant (*X*^2^ unadjusted *P*=0.04).”

“Most significantly, the rs7174027*A SNP flanking the *OCA2* gene promoter region/transcription start site was absent in AHM patients, compared to a frequency of 7.1% in the PM group (*X^2^* unadjusted *P*=0.0005) and 9.43% frequency in the BNMS control group (*X^2^* unadjusted *P*=8x10^-5^).”

**Supplementary materials have track changes and highlight marks that need to be removed.**

These are now removed in the revised Tables.

**If you would like to make changes to your financial disclosure, please include your updated statement in your cover letter.**

There are no changes to the authors financial disclosures.

**Journal Requirements:**

**1. Please ensure that your manuscript meets PLOS ONE's style requirements, including those for file naming. The PLOS ONE style templates can be found at
https://journals.plos.org/plosone/s/file?id=wjVg/PLOSOne_formatting_sample_main_body.pdf and**
[**https://journals.plos.org/plosone/s/file?id=ba62/PLOSOne_formatting_sample_title_authors_affiliations.pdf**](https://journals.plos.org/plosone/s/file?id=ba62/PLOSOne_formatting_sample_title_authors_affiliations.pdf)

This is now done.

**2. Please provide additional details regarding participant consent. In the ethics statement in the Methods and online submission information, please ensure that you have specified whether consent was informed.  If your study included minors, state whether you obtained consent from parents or guardians.**

In the Methods section this now reads,

“This study was approved by the Human Research Ethics Committee of Princess Alexandra Hospital (approval HREC/09/QPAH/162) and The University of Queensland (approval #2009001590) and conducted in accordance with the Declaration of Helsinki. Participants provided written informed consent and parental/guardian consent was also provided for those under 18 years.”

**3. Thank you for stating the following in the Competing Interests section: "I have read the journal's policy and the authors of this manuscript have the following competing interests: HPS is a shareholder of MoleMap NZ Limited and e-derm consult GmbH, and undertakes regular teledermatological reporting for both companies. HPS is a Medical Consultant for Canfield Scientific Inc. and MetaOptima Technology Inc., a Medical Advisor for First Derm, and has a Medical Advisory Board Appointment with MoleMap NZ Limited. The other authors have no conflicts of interest to declare."**

**Please confirm that this does not alter your adherence to all PLOS ONE policies on sharing data and materials, by including the following statement: "This does not alter our adherence to  PLOS ONE policies on sharing data and materials.” (as detailed online in our guide for authors http://journals.plos.org/plosone/s/competing-interests).  If there are restrictions on sharing of data and/or materials, please state these. Please note that we cannot proceed with consideration of your article until this information has been declared.**

This is now declared in the Competing Interests section.

"This does not alter our adherence to PLOS ONE policies on sharing data and materials.”

**Please include your updated Competing Interests statement in your cover letter; we will change the online submission form on your behalf.**

Now done.

**Please know it is PLOS ONE policy for corresponding authors to declare, on behalf of all authors, all potential competing interests for the purposes of transparency. PLOS defines a competing interest as anything that interferes with, or could reasonably be perceived as interfering with, the full and objective presentation, peer review, editorial decision-making, or publication of research or non-research articles submitted to one of the journals. Competing interests can be financial or non-financial, professional, or personal. Competing interests can arise in relationship to an organization or another person. Please follow this link to our website for more details on competing interests: http://journals.plos.org/plosone/s/competing-interests**

Understood.

**Review Comments to the Author**

**Reviewer #1: In the research article entitled “Albinism variants in individuals with amelanotic/hypomelanotic melanoma: increased carriage of TYR and OCA2 variants and their somatic mutation in AHM tumor tissue”, the authors use whole exome sequencing to look for rare variants (<1% MAF) in albinism-associated genes in a small group of patients who had amelanotic or hypomelanotic melanoma. They show that rare variants in TYR (OCA1) are more common in AHM than PM patients. No rare variants were found in OCA2,3,4,6,7 in the AHM patients but they did find higher incidence of a variant (p.V443I) in OCA2 that is below <1% in gnomAD but not in their own control group. They then analyzed blue eye color-associated polymorphisms at OCA2/HERC2 and found a significant difference in rs7174027*A (associated with darker eye color) between AHM (not detected) and PM (also controls). Lastly, the authors suggest that AHM could occur due to LOH or somatic mutation of the functional copy of TYR or OCA2. The authors support this model using TCGA melanomas which found higher incidence of mutations or CNV in albinism genes in amelanotic melanomas (n=7) compared to pigmented melanomas (n=431).**

We thank the reviewer for understanding and succinctly summarising the paper. **The authors have sufficiently responded to and corrected for previous reviewer’s comments.**

This modified manuscript has been through two cycles of revision before being considered by PLOS One. We thank the reviewer for considering that we have sufficiently responded to the PLOS Genetics reviews. **Minor Issues

There were 52 unknown pigment status melanomas sequenced as described in the text (line 182-185) and Figure S1 which were then not mentioned for the rest of the paper. Please remove this or clarify if and how these melanomas were used in this article.**

Figure S1 is the flow diagram of the 1233 BNMS participants to show how they were parsed in the analysis performed for this study. There were 581 melanoma cases with 383 individuals for whom WES was obtained. The genotype data for the 52 melanoma samples of unknown pigmentation status was included in the analysis presented in the Tables i.e.

Table 1, and Table S1 to S3 have a sub heading under Melanoma Cases “Total Melanoma cases N (MAF%) Total =581(WES =383)”.

Table 2 has the sub heading under Controls versus any melanoma cases, “BNMS Any Cases”

Note we have revised Table S2 to be the same format for the headings as Table 1, S1 and S3.

We do not feel it is required to restate that 52 of the total melanoma samples that WES were of unknown pigmentation phenotype, it is sufficient to say that they are included in the Total or any melanoma cases in the headings to the Tables.

**Due to the low number of amelanotic melanomas analyzed from TCGA melanoma dataset, the conclusions drawn from these seven tumors, while intriguing, are suggestive and inclusion of “and their somatic mutation in AHM tumor tissue” in the title could be misleading. Please consider revising your title to not over state these findings.**

We have changed the title to,

“Germline and somatic albinism variants in amelanotic/hypomelanotic melanoma: increased carriage of *TYR* and *OCA2* variants” **Small clarifications / fixes

Lines 221 to 223: Here the authors start with 18 variants and remove the two high frequency gnomAD variants which would leave 16 variants but the next sentence talks about the 15 rare variants. Would like a sentence explicitly stating that the p.V443I variant is common in your set in between these two sentences.**

This now reads,

“There were 18 coding variants in *OCA2* (Table S1, gnomAD MAF), two of which, p.R305W (5.05%) and p.R419Q (6.5%), have been described previously as being common [10], with a third variant p.V443I at 1.09% in MGRB controls (Table 1 and S1). Seven of the remaining 15 rare variants (<1%) have been reported as causative for OCA2 albinism …” **In Figure 1, please label the directionality of the OCA2 and HERC2 genes or label the haplotype region reference in line 306.**

The direction of gene transcription is now indicated above the gene names in Figure 1.

**There are different fonts being used in Table 3.**

This is now corrected

**There is a discrepancy on line 342, which states 463 PMs, and the footnote of Table 5, which states 431 PMs, for the TCGA analysis. Please resolve this discrepancy.**

The issue is that only 431 of the genotypes are available for the 463 PM samples. The footnote has been changed to read,

“… in 2/7 AHM (28%), 13/431 PM (3.02%) and 364/9732 other cancers (3.7%), where genotypes are available. …”

**The results section starting at line 337 has multiple duplicate words throughout.**

The use of a number of duplicate words has been reduced.

**Reviewer #2: A manuscript by Rayner and co-authors entitled “Albinism variants in individuals with amelanotic/hypomelanotic melanoma: increased carriage of TYR and OCA2 variants and their somatic mutation in AHM tumor tissue” may potentially be of interest to PLOS One readers, but the way the authors presented their research and organized the data in the manuscript is very difficult to follow and understand.**

We appreciate that there is difficulty in following the text, this is bought on by using both Illumina and WES genotyping methods to report variants in our collection. This requires some explanation for how the numbers of variants are detected and then analysed using the cut off of 1% variant allele frequency. Notably the OCA2 V443I allele is slightly above this cut off and was analysed separately (see comment and revision in response to reviewer 1).

**In the RESULTS, Analysis of albinism gene alleles in PM and AHM cases section the authors write:**

**Three of these TYR rare variants**

**202 (p.A23T, p.T373K and p.P460L) were found in the AHM population in comparison to six
203 (p.R217Q, p.V275F, p.R299H, p.N371T, p.T373K, p.P460L) in the larger PM group. Overall,
204 rare TYR variants were identified in 4.67% of AHM cases compared to 1.76% of PM cases, and
205 1.14% of MGRB controls (Table 1).**

**It is unclear how the value “4.67% of AHM cases” was calculated. As the sentence is written, one may believe that the authors sequenced 28 AHM cases and found 3 rare TYR variants in the patients. However, 4.67% of 28 AHM cases would be (28/100)*4.67=1.3076. Based on Table1 (column5 X row3), one wonders if the value 4.67 is meant to be understood as MAF (Minor Allele Frequency). The way it’s calculated, however is not commonly understood as MAF.**

In the original Table 1 the row title was “Sum *TYR* (MAF%) observed rare alleles (<1%) assayed”

and the equivalent original Table S1 row title was “Sum (MAF% ^s^) observed rare alleles *OCA1/TYR* (<1%)”, this indicated Minor Allele frequency, not as the reviewer has calculated it.

^s^ Genotypes calculated as “missing completely at random” with respect to disease status and % then appropriately weighted for each denominator of the variants being summed.

We apologise for the miswording in the text. This is now changed to read,

“Overall, rare *TYR* variants were at a MAF of 4.67% in AHM cases compared to 1.76% in PM cases, and 1.14% in MGRB controls (Table 1 and Table S1).”

**Data in the tables are poorly organized and difficult to understand. For instance, in Table 1, the same column contains different types of variables: the intersection of column “MGRB Control; N (MAF%); Total =1144; WES” with row 2 “Total observed rare alleles TYR (<1%) assayed” shows a value of “12 of 18”; and the intersection of the same column with row 3 “Sum TYR (MAF%) observed rare alleles (<1%) assayed” shows a value of “26 (1.14)”.**

We have tried to clarify this changing the title of row 2 to,

“**Allelic spectrum for *TYR* (MAF<1%) in each subgroup”**

The title of row 3 is now,

**“Total number of *TYR* alleles (MAF(<1%)) observed in each subgroup”**

These changes are now also made throughout the rest of the Tables for consistency.

**It’s unclear why p-values for statistical tests are placed in the row called “Sum TYR (MAF%) observed rare alleles (<1%) assayed.”**

The heading to the last three columns of this row was

Statistical Tests (*P*-value)

In the revised Table it now appears as

“SMMAT Tests (*P*-value)”

And below it is

“*X^2^* Statistical Tests (*P*-value)”

As has been explained, this is now explicit in each of the Tables.

**It is not immediately clear what the difference is between “Total observed rare alleles TYR (<1%) assayed” and “Sum TYR (MAF%) observed rare alleles (<1%) assayed”. The table headers should be concise and clear and the table legend should explain the headers if they are not self-evident.**

Total observed rare alleles now becomes “Allelic spectrum …” as explained above

And

Sum TYR (MAF%) … becomes “Total number of …” as explained above and consistent for all tables.

**One observes similar issues with Table 2. The way the data is organized is difficult to follow, the column and row headers are poorly labeled and explained.**

We have merged cells where appropriate to indicate which rows contrast the P-values we are testing.

**In addition, there are cells that the authors forgot to fill in. For example, what are the Total N(%) values for TYR in “Controls versus AHM cases”?**

These are now entered, apologies it was an oversight. **Additionally, data presented in the table are inconsistent. For example: columns 2 and 7 “Total N(%)” should contain percent values in parentheses in every cell.**

We have removed the N (%) from the column titles of Table 2.

**Table 3: references used in the footnotes, e.g. “Lek et al., 2016 [18]” and “Zhang et al., 2018 [46],” do not match the list of references. One observes the same problem with Supplementary Table S1. Omissions like these do not increase the reader’s confidence in the quality of the manuscript.**

Now corrected.

**Overall, the manuscript is full of omissions, mistakes, poorly organized content and is not clearly written. With this low quality of presentation, it is difficult to evaluate the true scientific value and merits of the research.**

The first reviewer was able to follow the text and appreciate the hypothesis and conclusions reached, but we agree it has not been easy to assimilate all the data in a clear manner. It would have been best to have WES done on all samples but this was not possible given the financial constraints and it was better to increase the genotype counts using the Illumina CoreExome data which was available for a superset.

As corresponding author I can be contacted at the following numbers and address:

Assoc. Prof Richard A. Sturm

Dermatology Research Centre

The University of Queensland Diamantina Institute (UQ-DI)

Level 5, Translational Research Institute (TRI)

37 Kent Street, Woolloongabba, Brisbane, QLD 4102, Australia

Tel.: 61 7 34437380

Email: [r.sturm@uq.edu.au](mailto:r.sturm@uq.edu.au)

I hope this report is found appropriate for re-review in *PLOS One.*

Sincerely yours,

Assoc. Prof. R.A. Sturm
